# Endoscopic Tumor Length Should Be Reincluded in the Esophageal Cancer Staging System: Analyses of 662 Consecutive Patients

**DOI:** 10.1371/journal.pone.0153068

**Published:** 2016-04-18

**Authors:** Michele Valmasoni, Elisa Sefora Pierobon, Alberto Ruol, Carlo Alberto De Pasqual, Gianpietro Zanchettin, Lucia Moletta, Renato Salvador, Mario Costantini, Stefano Merigliano

**Affiliations:** 3^rd^ Surgical Clinic - Center for Esophageal Disease, Department of Surgical, Oncological and Gastroenterological Sciences, University Hospital of Padova, Padova 35128, Italy; Queen Mary Hospital, HONG KONG

## Abstract

Esophageal cancer represents the 6^th^ cause of cancer mortality in the World. New treatments led to outcome improvements, but patient selection and prognostic stratification is a critical aspect to gain maximum benefit from therapies. Today, patients are stratified into 9 prognostic groups, according to a staging system developed by the American Joint Committee on Cancer. Recently, trying to better select patients with curing possibilities several authors are reconsidering tumor length as a valuable prognostic parameter. Specifically, endoscopic tumor length can be easily measured with an esophageal endoscopy and, if its utility in esophageal cancer staging is demonstrated, it may represent a simple method to identify high risk patients and an easy-to-obtain variable in prognostic stratification. In this study we retrospectively analyzed 662 patients treated for esophageal cancer, stratified according to cancer histology and current staging system, to assess the possible role of endoscopic tumor length. We found a significant correlation between endoscopic tumor length, current staging parameters and 5-year survival, proving that endoscopic tumor length may be used as a simple risk stratification tool. Our results suggest a possible indication for preoperative therapy in early stage squamocellular carcinoma patients without lymph nodes involvement, who are currently treated with surgery alone.

## Introduction

Worldwide, esophageal cancer accounts for more than 400,000 deaths every year. Despite recent improvements in survival esophageal cancer remains one of the deadliest diseases, with an overall 5-year survival less than 20% [1–5].

Prognostic stratification of these patients is crucial to provide them with the best multimodal treatment available. Nowadays this stratification is based on the TNM system developed by the American Joint Committee on Cancer (AJCC); it is based on the Tumor depth of invasion (T), lymph Node status (N), presence of Metastases (M), tumor grading and, only for squamocellular cancer, the location of the tumor within the esophagus [6–8].

Disease staging is based on endoscopy and Computed Tomography (CT) scan, and often integrated with Positron Emission Tomography—Computed Tomography (FDG-PET-CT scan) and endoscopic ultrasonography (EUS); those exams are not always available and are not always so accurate. Esophageal endoscopy, used routinely to diagnose esophageal malignancies, is a simple exam, which is well standardized and usually available even in community hospitals and in low-income socioeconomic settings [9–16].

Historically, endoscopic length of the tumor was a staging parameter in the TNM system but was subsequently abandoned in the 1987 version favoring tumor depth of invasion [17]. Lately though, various authors posed their attention again to the prognostic role of tumor length; likewise, tumor measures represent an important staging variable in many other cancers. Recently, several studies have identified a possible role for this parameter in the prognostic stratification of esophageal cancer. Some studies focused on the endoscopic length and other on the length measured on the pathological specimen; some studies were conducted on squamous cell carcinoma (SCC) and others on adenocarcinoma (AC) [18–31].

The present study aims to investigate the role of endoscopic tumor length (ETL) as a prognostic factor in esophageal cancers (SCC and AC), through the analysis of a consistent study cohort staged and treated at one single Center.

## Methods

All methods were carried out in accordance with approved guidelines. The study was approved by the Research Committee of the Department of Surgical, Oncological and Gastroenterological Sciences—University of Padova.

### Patients

Study cohort was selected by analyzing a database of 5,636 patients treated for esophageal cancer and prospectively collected at our Center from 1983 to 2014. Written informed consent was obtained for all patients enrolled in the database; this consent procedure was approved by our Research Committee. We selected all patients suitable for curative resection who underwent R0 esophagectomy (Ivor Lewis or Mckeown procedure [32–35]) for SCC or AC of the esophagus; from this initial pool we excluded all patients who received preoperative chemo and/or radiotherapy in order to avoid a confounding bias on the pathological result, those with metastatic disease found during surgery and patients deceased within 2 months after surgery. Each selected patient’s clinical record was reviewed to double check dubious or missing data. All patients for whom the required variables for our study were not available were excluded. All patients were studied before surgery with endoscopy, contrast swallow radiograms and CT scan [10].

### Data collection

The variables analyzed for the study were: demographics of patients (age, gender), pathologically determined T (pT) status, pathologically determined N (pN) status, endoscopic length of the tumor (ETL, defined as the total length of the lesion found on endoscopy and measured at our Center by equally trained endoscopists), localization of the primary tumor, histologic type, grading, follow-up after surgery. The TNM stage of disease was classified according to the AJCC 7^th^ version [6], even for pre 7^th^ version patients, reviewing the required parameters. See S1 Table for study data.

### Statistical analysis

We analyzed the cohort globally and then divided into two different groups based on histological type: SCC Group (squamous cell cancer) and AC Group (adenocarcinoma).

Cohort size allowed an additional subdivision of both SCC and AC groups based on TNM stage grouping according to AJCC 7^th^ version to analyze the influence of ETL on these different prognostic classes (Subgroup TNM 0-II that includes stages 0, IA, IB, IIA, IIB and subgroup TNM III that includes stage IIIA, IIIB and IIIC; TNM IV stage is not present in our cohort since it represents metastatic disease, a criteria of exclusion).

**Fig 1:**
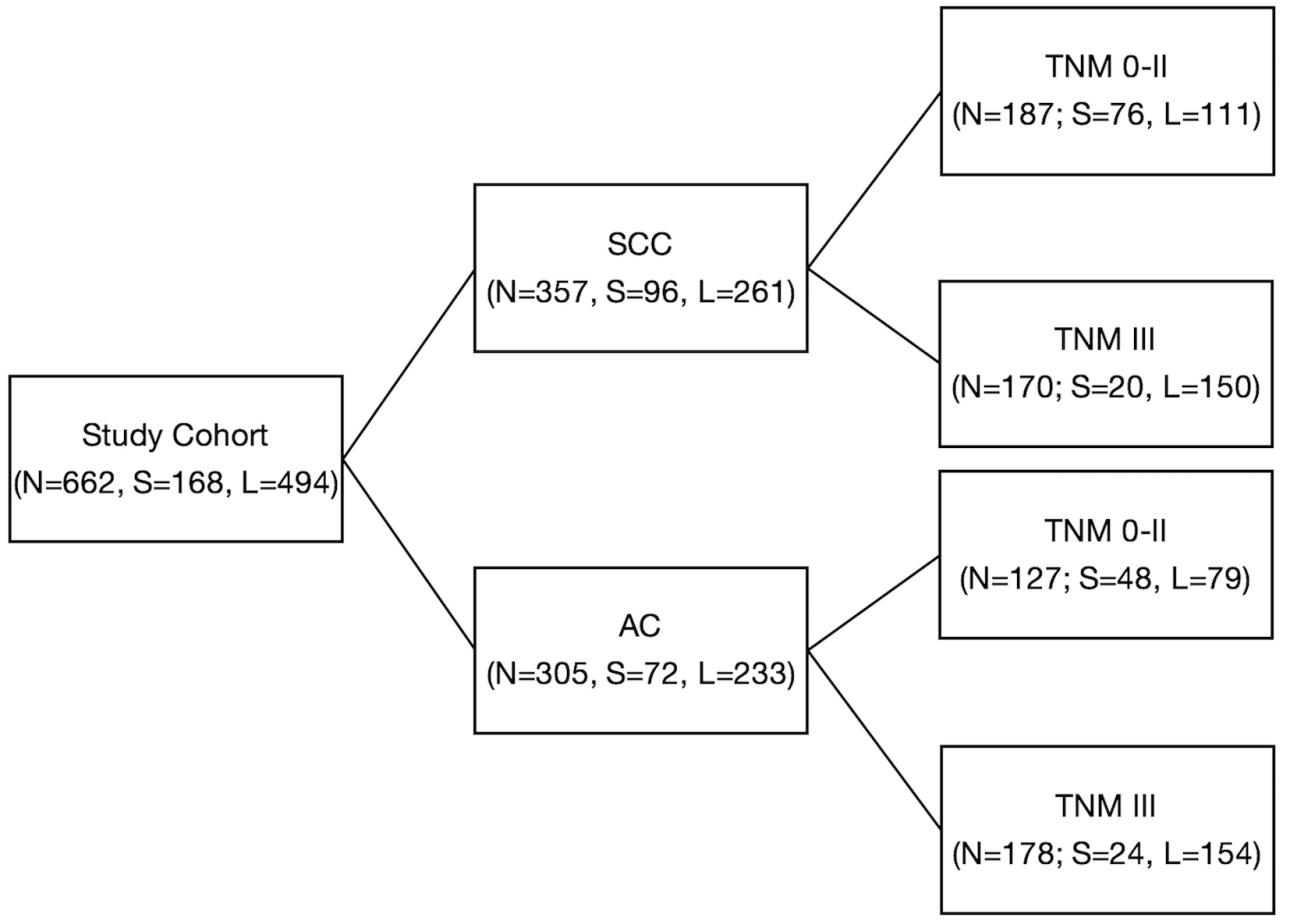
Study cohort with groups and subgroups. N: number of patients; S: Endoscopic Tumor Length < 3 cm; L: Endoscopic Tumor Length ≥ 3 cm; SCC: squamous cell carcinoma; AC: adenocarcinoma.

Optimal cutoff of ETL was identified with a regression tree survival analysis, comparison between survival curves plotted for ETL intervals of 1 cm and the literature review. Patients, overall and in the groups and subgroups, were then analyzed based on the ETL cutoff (S, short tumor; L, long tumor), (Fig 1).

Descriptive results are shown as mean ± standard deviation (SD) for the continuous variables and as size and frequency for categorical variables. Correlation between variables was evaluated using Pearson chi-squared test and Cox Logistic Regression.

Univariate survival analysis was performed with Kaplan-Meier statistic and differences between curves were calculated using log rank test. Cox proportional hazard model was used for the multivariate analysis. P value < 0.05 was considered significant. JMP software version 12.0.1 for Mac OS X (SAS Institute Inc.) was used for all statistical analyses.

## Results

The study cohort comprised 662 patients; 357 were treated for squamous cell cancer (SCC group) and 305 for adenocarcinoma (AC group). Table 1 shows demographic, clinical and pathological data. SCC and AC groups were further divided into subgroups according to the TNM staging system (Fig 1).

**Table 1:** Cohort demographics, clinical and pathological characteristics.

	Study Cohort (N = 670)	
	SCC Group (N = 357)	AC Group (N = 305)
**Age** [yrs, mean ± st. dev.]	62 ± 9.3	63 ± 11.2
**Sex** [N (%)]		
M	272 (76%)	276 (90%)
F	85 (24%)	29 (10%)
**Tumor Location**		
Upper	90 (25%)	0 (0%)
Middle	147 (41%)	6 (2%)
Lower/Cardia	120 (34%)	299 (98%)
**Tumor Grading** [N (%)]		
G1	83 (23%)	45 (15%)
G2	209 (59%)	173 (57%)
G3	65 (18%)	87 (28%)
**pT**		
Tis-1	65 (19%)	42 (14%)
T2	66 (18%)	54 (18%)
T3	172 (48%)	185 (61%)
T4	54 (15%)	23 (7%)
**pN**		
Negative	175 (49%)	103 (34%)
Positive	182 (51%)	202 (66%)
**pTNM** (AJCC)		
0-II	187 (52%)	127 (42%)
III	170 (48%)	178 (58%)
**ETL** [cm, mean ± st. dev.]	49.6 ± 25	54.5 ± 26.8
ETL < 3 cm	96 (27%)	72 (24%)
ETL ≥ 3 cm	261 (73%)	233 (76%)

N: number of patients; ETL: endoscopic tumor length; SCC: squamocellular carcinoma; AC: adenocarcinoma, pT: pathological tumor depth, pN: pathological lymph node involvement, pTNM: pathological prognostic stage according to American Joint Committee on Cancer 7^th^ Ed. (AJCC).

### Endoscopic tumor length cutoff and correlation with pT, pN and survival time

Mean ETL was 49.6 mm (Standard Deviation—SD 25) in the SCC group and 54.5 mm (SD 26.8) in the AC group.

In order to determine the optimal ETL cutoff, we performed a regression tree survival analysis; the best cutoff resulted to be 30 mm; so according to this value, tumors were classified as Short tumors (S: ETL < 30 mm) and Long tumors (L: ETL ≥ 30 mm).

**Fig 2:**
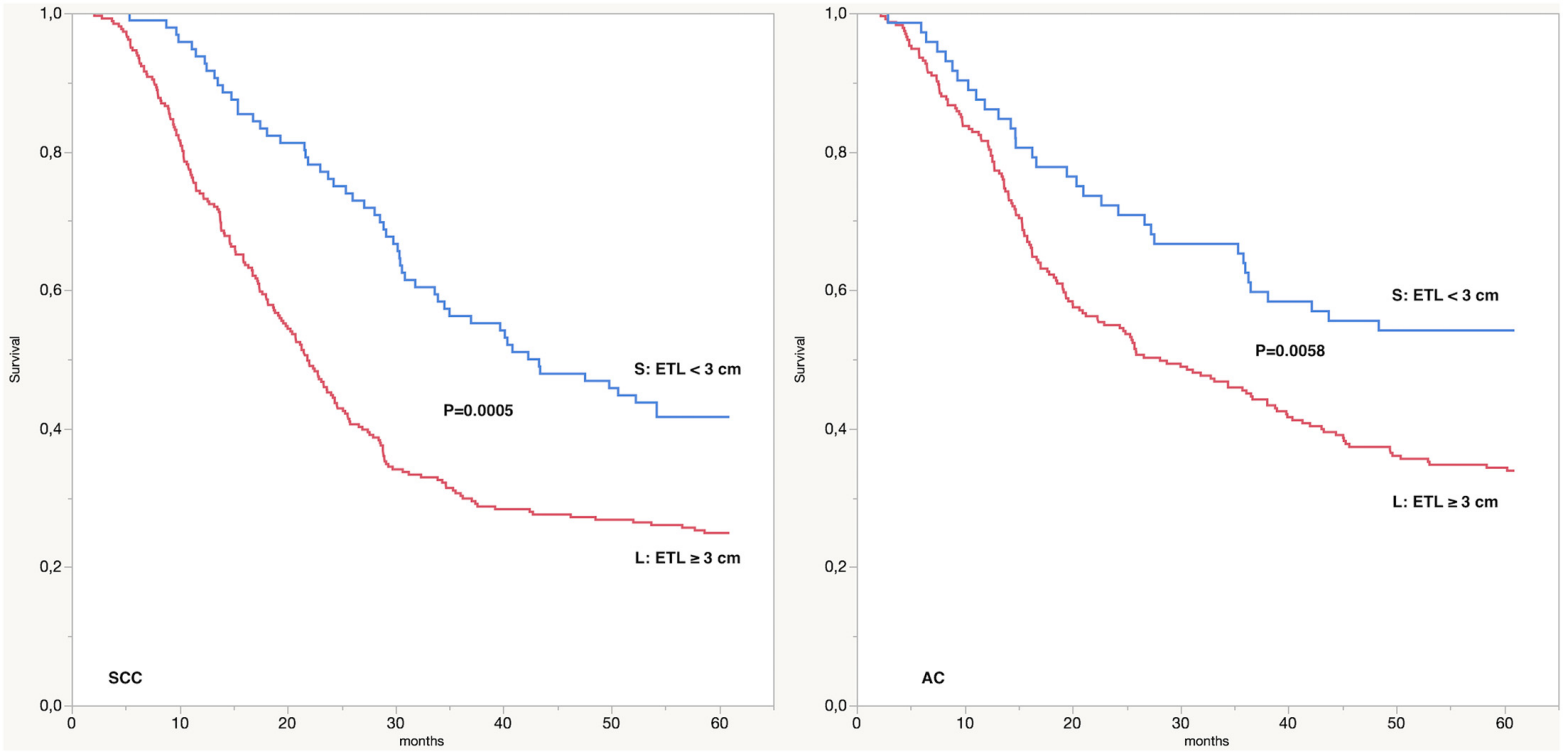
5-year survival according to endoscopic tumor length (ETL). SCC: squamocellular carcinoma; AC: adenocarcinoma; S: ETL < 3 cm; L: ETL ≥ 3 cm.

The correlation analysis between the ETL (considered as a continuous variable) and the pT and pN stage is statistically significant both in the SCC group (P<0.0001 and P = 0.0068 for pT and pN, respectively) and in the AC group (P<0.0001 and P<0.0001 for pT and pN, respectively). Likewise, the contingency analysis between the ETL as a categorical variable (S: ETL < 30 mm and L: ETL ≥ 30 mm) and the pT and pN is statistically significant (P<0.0001 in the SCC group both for pT and Pn; P<0.0001 in the AC group both for pT and pN). Bivariate analysis shows a significant inverse relationship between the ETL (as a continuous variable) and the 5-year survival in both groups (P = 0.0005 and P = 0.0058 for SCC and AC, respectively); this correlation was confirmed also considering the ETL as a categorical variable (S: ETL < 30 mm and L: ETL ≥ 30 mm), (Fig 2).

### Survival analysis

Five-year survival curves in the two groups SCC and AC, stratified according to the TNM stage, are consistent with the literature [5, 6, 32, 35].

Kaplan-Meier univariate analysis, showed that patients with ETL < 3 cm have a 5-year survival rate significantly better compared to those with ETL ≥ 3 cm (47% versus 29.1%, log-rank P<0.0001, respectively); this result is confirmed also in the SCC and AC groups (SCC: 41.6% versus 24.9%, log-rank P<0.0001, respectively; AC: 54.1% versus 33.9%, log-rank P = 0.0030, respectively). Table 2 shows the results of the Kaplan-Meier univariate survival analysis for tumor location, tumor grading, pT, pN and ETL.

**Table 2:** Univariate 5-year survival analysis.

	5-year Survival (%)			
	SCC Group (N = 357)	P	AC Group (N = 305)	P
**Tumor Location**		0.0626		NC
Upper	28.8		NC	
Middle	33.3		NC	
Lower/Cardia	25		NC	
**Tumor Grading**		0.0418		<0.0001
G1	40.9		60	
G2	26.3		39.8	
G3	24.6		22.2	
**pT**		<0.0001		<0.0001
T is-2	45		62.5	
T 3–4	20.3		27.7	
**pN**		<0.0001		<0.0001
Negative	43.4		43.4	
Positive	15.9		15.9	
**ETL**		<0.0001		0.0030
ETL < 3 cm	41.6		54.1	
ETL ≥ 3 cm	24.9		33.9	

SCC: squamocellular carcinoma; AC: adenocarcinoma; ETL: endoscopic tumor length; pT: pathological tumor depth; pN: pathological lymph node involvement; NC: Tumor location not considered for AC, according to actual TNM staging system.

**Fig 3:**
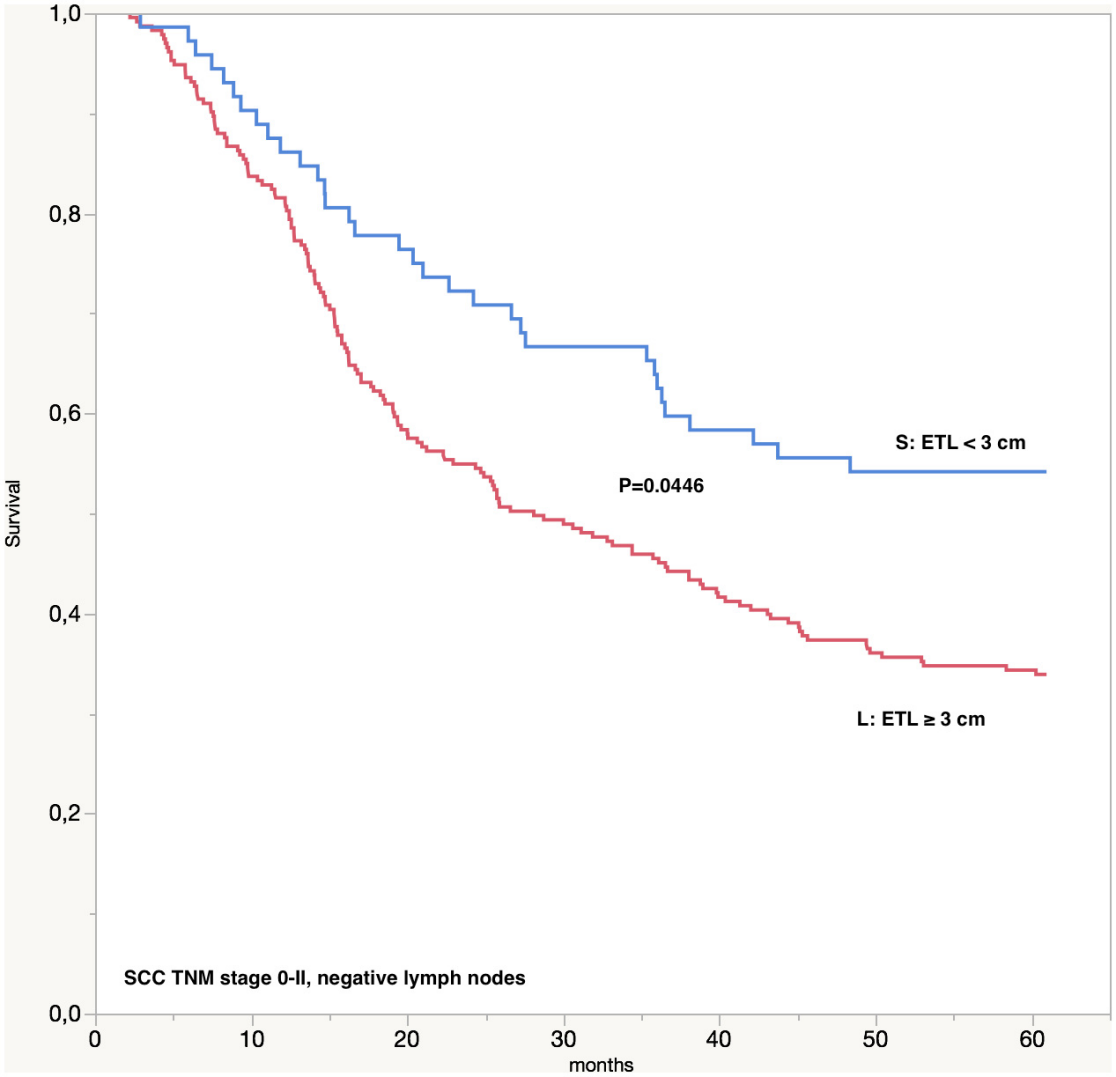
5-year survival for stage 0-II SCC patients, with negative lymph nodes, according to endoscopic tumor length (ETL). SCC: squamocellular carcinoma; ETL: endoscopic tumor length; S: ETL < 3 cm; L: ETL ≥ 3 cm.

Survival analysis in the TNM subgroups showed a significant and independent prognostic value of ETL only in the SCC/TNM stage 0-II subgroup (5-year survival rate for short tumors 47.3% versus 37.8% for long tumors, log-rank P = 0.0342). Moreover, we analyzed the effect of the ETL on the survival of SCC patients according to the lymph node status: in patients without lymph node involvement (pN0), a significant difference was found between short tumors and long tumors (5-year survival rate 51.5% versus 38.7%, log-rank P = 0,0210, respectively); a significant difference was found also in patients with lymph node involvement (pN+) between short tumors and long tumors (5-year survival rate 21.8% versus 14.6%, log-rank P = 0.0330, respectively). Analysis of SCC subgroups showed a significant difference only in the TNM 0-II subgroup without lymph node involvement (pN0) between patients with short tumors and patients with long tumors (5-year survival rate of 52.3% versus 41.3%, log-rank P = 0.0446, respectively), (Fig 3).

### Multivariate Survival Analysis

A Cox proportional hazard model was evaluated entering the TNM system variables (pT, pN, grading and tumor localization) and ETL for the SCC and AC group separately. Multivariate analysis results are shown in Table 3. ETL reached statistical significance only in the SCC group (HR 1.47; 95% CI 1.08–2.03; P = 0.0132).

**Table 3:** Multivariate analysis.

	SCC (N = 357)			AC (N = 305)		
	HR	95% CI	P	HR	95% CI	P
**Tumor Location**			0.0673			NC
**Upper**	1.35	0.97–1.85		NC		
**Middle**	1.00	-		NC	NC	
**Lower**	1.36	1.01–1.83		NC	NC	
**Tumor Grading**			0.1003			0.1854
**1**	1.00	-		1.00	-	
**2**	1.34	0.97–1.89		1.08	0.65–1.88	
**3**	1.48	0.99–2.21		1.43	0.83–2.56	
**pT**			0.0009			0.0451
**is-2**	1.00	-		1.00	-	
**3–4**	1.68	1.23–2.31		1.50	1.00–2.29	
**pN**			0.0001			<0.0001
**Negative**	1.00	-		1.00	-	
**Positive**	1.71	1.29–2.27		3.49	2.02–5.48	
**ETL**			0.0132			0.8685
**< 3 cm**	1.00	-		1.00	-	
**≥ 3 cm**	1.47	1.08–2.03		1.03	0.70–1.56	

SCC: squamocellular carcinoma; AC: adenocarcinoma; ETL: endoscopic tumor length; pT: pathological tumor depth; pN: pathological lymph nodes involvement; NC: Tumor location not considered for AC according to actual TNM staging system.

## Discussion

In our series, ETL correlates significantly with pT and pN, hence with survival. Moreover, our analysis underlines ETL as an independent prognostic factor that allows a better stratification of patients with early stages (TNM stage 0-II) esophageal SCC.

Esophageal cancer is an aggressive tumor, often diagnosed at advanced stages, characterized by an overall 5-year survival rate of less than 20% [1–5]. An accurate staging system allows to identify those patients who are suitable for surgery alone, or who need preoperative chemoradiotherapy.

The presence of lymph node metastasis is an indication for preoperative therapy, even in early tumor stages according to the current guidelines [5, 35, 36]. CT scan together with FDG-PET-CT scan and EUS plays a significant role in TNM stage allocation; however, FDG-PET-CT scan, useful to determine unknown metastasis, has a low accuracy in detecting locoregional metastatic lymph nodes, while EUS, even if more reliable in detecting locoregional metastatic lymph nodes, it is operator dependent and not always routinely available [9–16].

ETL and circumferential involvement were abandoned by the AJCC as a prognostic stratification parameter starting from the 1987 TNM staging version [17]. However, more recently many authors studied the possible role of the tumor length, either clinical or pathological, in the stratification of esophageal cancer prognosis.

Endoscopy is the first diagnostic exam to be performed in all patient with esophageal cancer and it is readily available in the vast majority of Centers; therefore, ETL is a standardized easy-to-obtain parameter. ETL also correlates significantly with pathologically measured tumor length as shown by Gaur et al and Wang et al. [19, 29]

The present study results highlight the possibility to obtain a simplified prognostic classification based on the endoscopic findings, given its significant correlation with pT and pN: in our series patients with tumors < 3 cm (both SCC and AC histotypes) showed a significantly better 5-year survival.

We chose 3 cm as the cutoff value for the ETL; to identify this value we conducted a regression tree survival analysis, testing endoscopic tumor length as a predictor of survival; considering that in the literature there is no agreement in terms of which is the best method to define cutoffs of this kind [37], we validated our result with survival curves analysis for different ETL intervals and took into accounts also the results published by other authors (Table 4).

**Table 4:** Recent tumor length studies identifying a prognostic significant cutoff.

Author, year	N. of patients	Histology	TL measured / Cutoff value	Method used to calculate cutoff
**This Study**	662	SCC / AC	Endoscopic / 3 cm	Regression Tree Model
**Mirinezhad, 2014**	71	SCC / AC	Pathological / 4 cm	ROC Curves
**Feng, 2013**	132 (> 70 yr old)	SCC	Specimen / 4 cm	ROC Curves
**Zeybek, 2013**	116	SCC / AC	Pathological / ≤3, 3–6, ≥6	-
**Wang, 2012**	244	SCC	Endoscopic / 4 cm	Regression Tree Model
**Song, 2012**	201	SCC	Pathological / 3 cm	Survival Analysis
**Wang, 2011**	582	SCC	Specimen / 3 cm	Survival Analysis
**Gaur, 2011**	164 + 109 (validation)	AC	Endoscopic / 2 cm	Regression Tree Model
**Bolton, 2009**	133	AC (pT1 only)	Pathological / 3 cm	Survival Analysis
**Yendamuri, 2008**	209	SCC / AC	Pathological / 3 cm	Survival Analysis
**Bolshweiler, 2006**	213	SCC / AC	Pathological / 3 cm	Survival Analysis
**Griffiths, 2006**	309	SCC / AC / Other	Pathological / 3.5 cm	Median value

TL: tumor length; SCC: squamocellular carcinoma; AC: adenocarcinoma.

Univariate analysis showed that ETL is a significant prognostic factor for patients with esophageal cancer (Table 2). Multivariate analysis confirmed ETL as an independent risk factor for SCC group (Table 3). We wanted to further analyze the role of ETL as an independent prognostic factor by grouping patients based on TNM stage in order to evaluate if the introduction of the tumor length parameter could lead to a better prognostic stratification regardless of the pT and pN status. Survival analysis confirmed that patients with ETL < 3 cm have a significantly better 5-year survival than patients with ETL ≥ 3 cm in the SCC/TNM 0-II subgroup. As a matter of fact, inside this subgroup, patients with tumors ≥ 3 cm have a significantly lower survival in comparison with patients with tumors < 3 cm even if the N status is negative. This result may possible be related to the lymphatic vessels invasion tendency (lymphangiosis) of SCC tumors as described by Stein et al. [38] and Cense et al. [39]

For the other SCC/TNM subgroups and for all the AC/TNM subgroups, we did not identify a significant independent prognostic role of ETL; in this case, the difference in terms of survival between the short and long tumors may be related to the significant correlation between ETL and both pT and pN status.

Wang et al. [19] studying 244 patients who underwent esophagectomy for SCC, showed that an ETL cutoff value of 4 cm is significantly correlated with pT, pN status and 5-year survival, and that it is a significant risk factor independent from pN.

Gaur et al. [29] from M. D. Anderson Cancer Center found that ETL cutoff value of 2 cm is significant at multivariate survival analysis of esophageal adenocarcinoma; we did not find a significance for ETL in adenocarcinoma and this may be due to the fact that we analyzed our cohort based on pathological and not clinical staging.

The study of Gaur et al. was based on a previous study from the same group in which Yendamuri et al. [27] stratified 209 SCC and AC patients according to pathological TNM stage and lymph node status; they found that the impact of pathological tumor length (cutoff 3 cm) on survival was significant in lower TNM stages (stages I-II) and in patients without lymph node involvement.

Furthermore, Song et al. [30] studied a cohort of 201 patients who underwent surgery for early esophageal SCC (pT1-2 pN0): patients with tumors ≤ 3 cm (pathological tumor length) had a significantly better survival than patients with longer tumors. Gaur and Song findings are in agreement with our results.

In our series tumor grading was not an independent prognostic factor both for SCC and AC tumors, even if it was statistically significant at univariate survival analysis in both groups. In the SCC group, also tumor location was not statistically significant (Tables 2 and 3). Situ et al., Hu et al. and Wijnhoven et al. reported similar results [40–43].

Our study has some limitations. First, this is a retrospective cohort study, with inherent study design limits; secondly, endoscopic length cannot be obtained for non-passable tumor stenosis. Some may consider dilation to pass through the stricture with the endoscope, but the procedure may be hazardous in terms of risk of perforation [15]; in light of this, ETL data of advanced stenotic lesions are missing and, even though we showed that ETL could be useful for early stages of disease, this represents a selection bias. All endoscopies and surgical interventions were carried out at our center by similarly trained endoscopists and surgeons, following the same protocols, and we are confident that this limit the possible biases related to multiple operators.

We have excluded patients submitted to neoadjuvant chemo/radiotherapy in order to avoid confounding factors on the correlation between endoscopic tumor length and pathological result; because of this selection criterion we cannot analyze how tumor length affects T, N and survival in patients undergone CT/RT.

Another potential bias is represented by the fact that the current TNM staging system classified gastro-esophageal junction tumors as esophageal tumors and so we did not analyze these lesions separately, even if there is some evidence in literature that they may have a different biological behavior [44]; further studies are required to clarify this aspect.

The advantages of this study are the large sample size, and that all patients have been studied and treated at the same institution, using uniform diagnostic and treatment protocols. We were able to conduct the study both in patients with SCC and AC, trying to clear the role of ETL in both major esophageal cancer histotypes.

In line with the other authors cited, we agree that prospective multicenter studies are needed to validate the already available results.

In conclusion, we showed that ETL plays a role as a prognostic factor in esophageal cancer. Our data suggest a possible benefit from preoperative treatment in early stages non cervical SCC N0 patients who, at the moment, are treated with surgery alone.

We strongly believe that ETL needs to be reassessed as a valuable stratification parameter in the forthcoming next TNM staging system revision (AJCC).

As a closing consideration, one strength of ETL as a prognostic variable is that it is easy to obtain also in poor socioeconomic area (where SCC of the esophagus is prevalent to adenocarcinoma [3, 5]) where it could be sufficient for an initial evaluation of the prognosis and therefore of the impact of the patient on the healthcare system.

## Supporting Information

S1 Table Minimal Data Set. Study data.
(XLSX)
